# Burial Environment Drives Seed Mortality of Kochia (*Bassia scoparia*), Wild Oat (*Avena fatua*), and Volunteer Canola (*Brassica napus*) Irrespective of Crop Species

**DOI:** 10.3390/plants10091961

**Published:** 2021-09-20

**Authors:** Charles M. Geddes

**Affiliations:** Agriculture and Agri-Food Canada, Lethbridge Research and Development Centre, 5403 1st Avenue South, Lethbridge, AB T1J 4B1, Canada; charles.geddes@agr.gc.ca

**Keywords:** demography, edaphic factors, integrated weed management, seedbank, seed fate, seed longevity, seed microsite, soil type, weed ecology

## Abstract

Models of weed population demography are critical to understanding the long-term viability of management strategies. The driving factors of weed seedbank persistence are often underrepresented in demographic models due to the cumbersome nature of seedbank research. Simplification of weed seedbank dynamics may induce substantial error in model simulations. A soil bioassay was conducted to determine whether growth of different crop species, including wheat (*Triticum aestivum* L.), canola (*Brassica napus* L.), and field pea (*Pisum sativum* L.), differentially impact seed mortality of kochia [*Bassia scoparia* (L.) A.J. Scott], wild oat (*Avena fatua* L.), and volunteer canola in seven burial environments in western Canada. Weed seed survival after the 7 week burial period varied widely among burial environments (from 8% to 88% when averaged among weed and crop species), whereas growth of the different crop species had negligible impact on seedbank persistence. Among environments, wild oat seed survived the greatest (79%), followed by kochia (20%), and volunteer canola (6%). Weed seed survival was associated with soil physical properties (texture) and seed microsite characteristics (temperature), but not crop species or soil chemical properties. Overall, these data support the need for greater integration of soil and environmental parameters into models of weed population demography.

## 1. Introduction

Herbicide-resistant weeds are a growing concern for farmers globally. Recent 2014–2017 estimates for the Canadian prairie region (comprising 87% of annual-cropped area in Canada) suggest that herbicide-resistant weeds infest about 9.6 M ha (35% of total area) of farmland under annual crop production, which has grown from 7.7 M ha (29%) in 2007–2009, and 1.0 M ha (4%) in 2001–2003 [1]. The most recent round of surveys estimated that herbicide-resistant weeds cost prairie farmers CAD 530 M annually in increased herbicide usage, and decreased crop yield and quality [1].

Kochia [*Bassia scoparia* (L.) A.J. Scott], wild oat (*Avena fatua* L.) and volunteer canola (*Brassica napus* L.) are among the most problematic weed species impacting prairie cropland in Canada. In a 2017 survey of 1232 annual-cropped fields in Alberta, CA, kochia, wild oat, and volunteer canola were the 15th, 5th, and 4th most abundant weeds present after post-emergence herbicide application [2]. Kochia was the most abundant weed species in the southern Mixed Grassland ecoregion of Alberta, and its northern extent in Canada is limited by thermal time requirements for seed production [2,3,4]. Wild oat and volunteer canola were abundant throughout the agricultural production area of Alberta [2].

Resistance to multiple herbicide sites-of-action exacerbates the impact of kochia, wild oat, and volunteer canola on Canadian farms by limiting the herbicide options available for their management [5,6,7]. In the Canadian prairies, resistance to 3, 4, and 3 herbicide sites-of-action have been documented in kochia, wild oat, and volunteer canola, respectively [8]. Kochia populations in Canada can exhibit resistance to acetolactate synthase (ALS) inhibitors [Herbicide Resistance Action Committee (HRAC) Group 2], synthetic auxins (HRAC Group 4), and glyphosate (HRAC Group 9) [8,9,10,11]. Wild oat has been reported with resistance to acetyl-CoA carboxylase inhibitors (HRAC Group 1), ALS inhibitors, very long chain fatty acid synthesis inhibitors (HRAC Group 15), and protoporphyrinogen oxidase inhibitors (HRAC Group 14) [8]. Volunteer canola can exhibit resistance to ALS inhibitors, glyphosate, or glufosinate-ammonium (HRAC Group 11) resulting from widespread production of herbicide-resistant canola cultivars in this region [12,13]. These volunteer plants return as problematic weeds in subsequent crops with similar herbicide resistance traits, such as soybean [14,15].

Proliferation of herbicide-resistant annual weeds warrants implementation of integrated weed management (IWM) strategies where non-chemical, cultural, mechanical, and biological tools are used in tandem with chemical weed management. Weed control programs may be augmented when management efforts target weeds at multiple different life-stages [16]. However, the majority of weed control tools implemented in the Canadian prairies target weeds at the seedling stage, often allowing weed escapes to complete their life cycles unhindered by a thorough IWM program.

The soil seedbank represents a critical life-stage in annual weed growth and development because all individuals in a population must pass through the seed life-stage, resulting in high elasticity of the population growth rate to targeted management efforts [17,18]. Kochia seeds exhibit little-to-no innate dormancy, resulting in rapid soil seedbank decline (after ~1–2 years) [19,20]. Volunteer canola, resulting predominantly from large canola seed losses at harvest [21], can enter secondary seed dormancy, resulting in a moderately persistent seedbank that persists in western Canada for 2–3 years on average [22,23,24]. Wild oat can exhibit both primary and secondary seed dormancy, typically resulting in 4–5 year seed longevity in the soil seedbank [25]. Modeling weed population demography can provide insight into the long-term efficacy of weed management practices. However, model accuracy is limited by the accuracy of data used for parameterization. Soil seedbank persistence data are often underrepresented or simplified in models of weed population demography, e.g., [26,27], due in part to the laborious and cumbersome nature of seedbank research. The response of belowground weed life-stages to management efforts represents a critical knowledge gap in weed life cycle analysis, and may manifest significant error in demographic model simulations.

Crop growth and development can impact weed seed germination and early seedling establishment through resource-limiting (direct) or non-resource-limiting (indirect) competition [28]. For example, moisture use by growing plants may limit seed microsites for safe germination and recruitment [29]. Alternatively, indirect interference from neighboring plants can alter seed germination and development though root exudation, residue decomposition, or volatilization of secondary metabolites (i.e., allelopathy) [30]. In a soil bioassay, aqueous extracts of winter wheat (*Triticum aestivum* L.) shoot tissue chemically suppressed germination of kochia seeds but not canola or wild oat [31]. However, these same extracts reduced radicle elongation of kochia and canola by 34% and 52%, respectively, but did not impact wild oat radicle development [31]. Therefore, interference from growing crops could alter longevity of weed seeds in the soil seedbank. The current study was designed as a preliminary assessment of the impact of wheat, canola, and field pea (*Pisum sativum* L.) crop growth and burial environment on seed mortality of kochia, wild oat, and volunteer canola in the soil seedbank.

## 2. Materials and Methods

### 2.1. Experimental Design and Treatment Structure

A 7 week bioassay experiment was conducted to determine the impact of crop growth on mortality of buried kochia, wild oat, and volunteer canola seeds in the soil seedbank. The bioassay followed a three-way factorial split plot randomized complete block design with three replicates. The entire experiment was repeated in seven different burial environments consisting of different soils and environmental conditions (Table 1 and Table 2). The three factors were crop species (3), weed seed species (3), and burial environment (7). Within each burial environment, the main plot factor consisted of crop species (wheat, canola, and field pea), whereas the split plot factor consisted of weed seed species (kochia, wild oat, and volunteer canola).

### 2.2. Experimental Logistics

The kochia and wild oat seeds were collected in 2019 from established field populations near Lethbridge, AB, CA (49.69° N, −112.76° W), and the volunteer canola was collected from harvest samples of canola grown in this same location. The seeds were dried at room temperature and cleaned using a Pfeuffer MLN sample cleaner (Pfeuffer GMBH, Kitzingen, BY, Germany). The cleaned seed accessions were stored at 4 °C until use.

A germination assay was used to assess seed viability for each weed species before experimental establishment in spring 2020. Fifty seeds from each species were added to separate 90 × 15 mm petri dishes (CA73370-010, VWR^®^ International, LLC) each fitted with two 90 mm filter papers (16924113, Grade 1, Whatman^®^). The seeds were imbibed in 8 mL diH_2_O, enclosed in a plastic Ziploc^®^ bag (SC Johnson & Son Inc., Racine, WI, USA) and placed in the dark. Germinated seeds were counted and removed every 2 days for a total of 14 days. Seeds were considered germinated when the radicle protruded through the seed coat. The seed germination assay was replicated four times for each weed species, and the number of germinated seeds was used to determine the percentage of viable seeds in each weed seed accession.

The seeds were buried in 10 cm diameter 500 µm nylon mesh envelopes (Industrial Netting, Maple Grove, MN, USA). Each envelope was filled with 100 viable seeds of a single weed species. One mesh envelope for each weed species was buried in each burial mesocosm at a depth of 10 cm. A 10 cm burial depth was chosen to represent the depth of a typical tillage pass in western Canada. The burial order of weed species was randomized within each mesocosm. Each mesocosm consisted of a 10 × 10 × 12 cm plastic pot with four 1 cm diameter holes in the bottom to facilitate water drainage. Each mesocosm was filled with a different field soil. Different soils were used for each burial environment (Table 1 and Table 2). The soils varied in collection location, predominant vegetation, and edaphic factors (Table 1 and Table 2). Ten seeds of either spring wheat ‘AAC Brandon’, canola ‘DKTF96SC’ (Bayer CropScience, Calgary, AB, CA), or field pea ‘CDC Meadow’ were planted in different mesocosms at depths of 3.8, 1.2, and 2.5 cm, respectively. Within each burial environment (Table 1), the mesocosms were placed in a randomized complete block design in an area with natural sunlight, and watered daily to field capacity. The mesocosms were fertilized at 2 and 4 weeks after planting with 25, 25, and 25 mg kg^−1^ of N, P, and K, respectively.

### 2.3. Data Collection

The main measurement consisted of the percentage of weed seeds that survived the 7 week burial period. The mesocosms were destructively sampled 7 weeks after planting, and the seed mesh envelopes were recovered by washing gently with water. The envelopes were opened, and the seeds remaining in each envelope were removed, washed gently, and patted dry with paper towel. Seed viability and therefore survival were determined using a seed pinch test [24,32]. Seed survival was expressed as a percentage of the number of viable seeds buried in the mesocosm at the beginning of the 7 week bioassay. Soil edaphic factors were evaluated at the beginning of the experiment (Table S1; Down to Earth Labs, Lethbridge, AB, CA). Soil temperature at 10 cm depth was recorded hourly using iButton^®^ temperature loggers (DS1921G-F5#, Embedded Data Systems, LLC, Lawrenceburg, KY, USA). Temperature data within each environment were used to determine the mean daily maximum (T_max_), minimum (T_min_), and average (T_avg_) soil temperature at 10 cm depth averaged over the 7 week burial period. The mean diurnal range in soil temperature (T_diurnal_) was determined by subtracting daily minimum from daily maximum temperatures and averaging over the 7 week burial period. Cumulative thermal time [Growing degree days (GDD, T_base_ 0 °C)] was determined for each burial environment using soil temperature at 10 cm depth following the methods outlined by Schwinghamer and Van Acker [33].

### 2.4. Statistical Analysis

The MIXED procedure in SAS 9.4 (SAS Institute, Inc., Cary, NC, USA) was used to analyze the weed seed survival data. The model followed a split plot randomized complete block design. The main and interaction effects of weed seed species, crop species, and burial environment were considered fixed factors, whereas experimental replication nested within burial environment, and the interaction of crop species and experimental replication nested within burial environment, were considered random factors. The UNIVARIATE procedure was used to assess residual normality using the Shapiro–Wilk test, whereas homogeneity of variance was assessed visually by plotting the residuals against the predicted values [34]. Proportional seed survival data were arcsine square root transformed and the covariance structure of residuals was grouped by weed species to meet the assumptions of normality and homoscedasticity. Outliers did not warrant removal. Variance component analysis was conducted with the type 3 sums of squares method to assess the percentage of total variance allocated to each factor. Means were separated according to Tukey’s HSD (α = 0.05).

The PLS procedure was used for partial least squares (PLS) analysis to extract underlying latent variables and identify the variables that were most influential and associated with seed survival based on the nonlinear iterative partial least squares algorithm [35]. Logarithm-transformed explanatory and response variables were centered and scaled prior to PLS analysis. The initial model contained 16 independent variables and 3 dependent variables. The independent variables included crop species, soil edaphic factors [including: concentrations of soil NO_3_-N, P, K, SO_4_-S, organic matter (OM) and soluble salts, pH, and sand, silt, and clay contents], and seed microsite characteristics (including: T_min_, T_max_, T_avg_, T_diurnal_, and GDD). The dependent variables included seed survival of kochia, wild oat, and volunteer canola. One-at-a-time cross validation was used along with the predicted residual sums of squares statistic and van der Voet’s statistic to optimize the number of factors extracted and avoid overfitting the model [36]. The model was pruned to contain only the variables that were most influential on variation in weed seed survival based on a variable importance for the projection (VIP) statistic ≥0.8 and assessment of the regression coefficients [36,37].

## 3. Results

### 3.1. Weed Seed Survival

**Figure 1:**
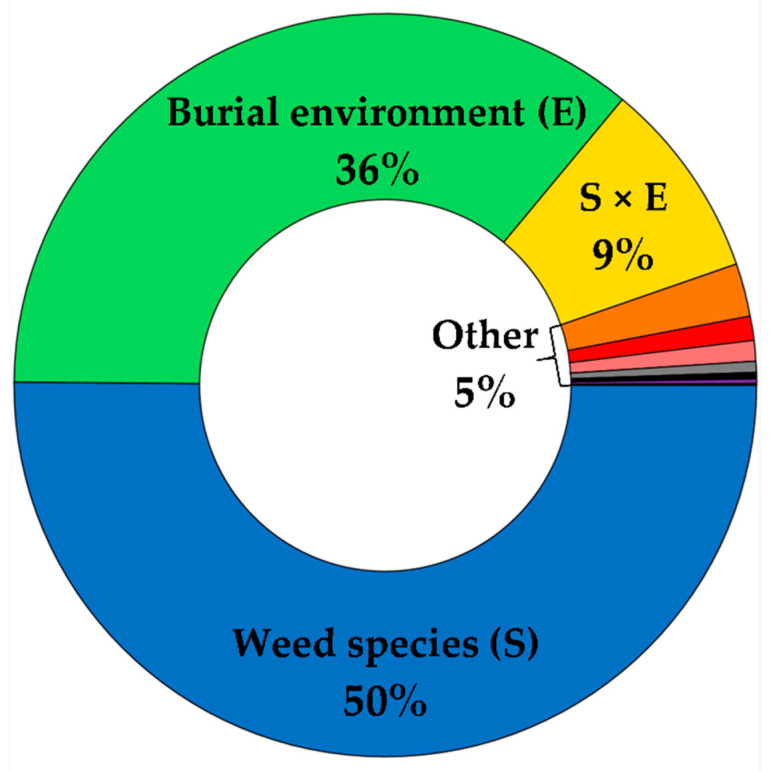
Percent of total variance allocated to each factor included in the linear mixed effects model for seed survival of kochia, wild oat, and volunteer canola in a 7 week bioassay experiment planted to wheat, canola, and field pea. Other includes all remaining fixed and random factors.

**Figure 2:**
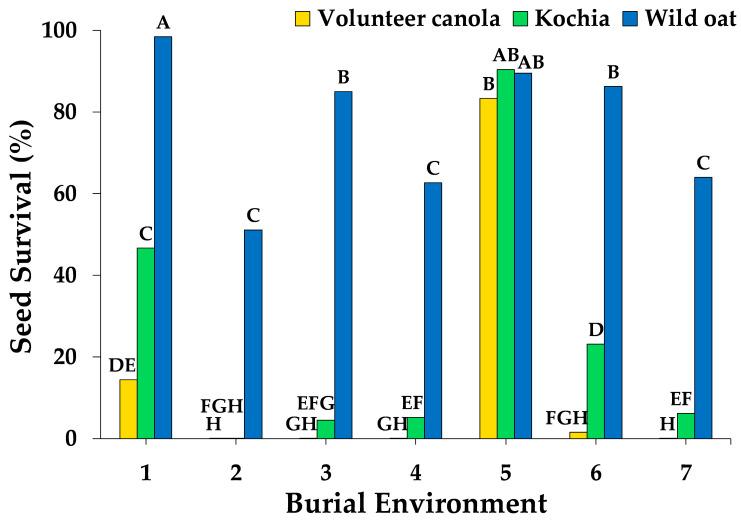
The percentage of volunteer canola, kochia, and wild oat seeds that survived a 7 week burial period in seven different environments planted to wheat, canola, and field pea (*n* = 9). Bars indicate back transformed arcsine square root means among crop species. Different letters indicate significant difference based on Tukey’s HSD (α = 0.05).

Weed seed survival was influenced by the weed species of interest (*p* < 0.0001) and burial environment (*p* < 0.0001), but was unaffected by growth of different crop species (*p* = 0.1898). Weed species, burial environment, and their interaction effect accounted for 50%, 36%, and 9%, respectively, of the variability in weed seed survival, whereas all main and interaction effects with crop species accounted for <3% (Figure 1). On average, the wheat had reached the mid- to late-tillering stage (BBCH 25) by the end of the burial period, whereas the canola and field pea were either finished (BBCH 71) or near-finished (BBCH 67) flowering, respectively (data not shown). Among environments, the greatest seed survival after the 7 week burial period was observed for wild oat (79%), followed by kochia (20%) and volunteer canola (6%) (Figure 2). Differences in burial environment resulted in wide variation in seed survival from 8% to 88% when averaged among weed species (Figure 2). There was a general trend among environments where a greater percentage of wild oat seeds survived compared with kochia and volunteer canola, with the exception of burial environment 5 in which similar seed mortality was observed for all three weed species (Figure 2). Near-complete (>99%) seed mortality of volunteer canola was observed in four of the seven environments, whereas >99% seed mortality of kochia was observed in burial environment 2 only (Figure 2). Together, these results suggest that soil edaphic factors and seed microsite characteristics drove weed seed mortality during crop growth and development, but the crop grown had minimal impact on seed longevity in the soil seedbank.

### 3.2. Contribution of Soil Edaphic Factors and Seed Microsite Characteristics

**Figure 3:**
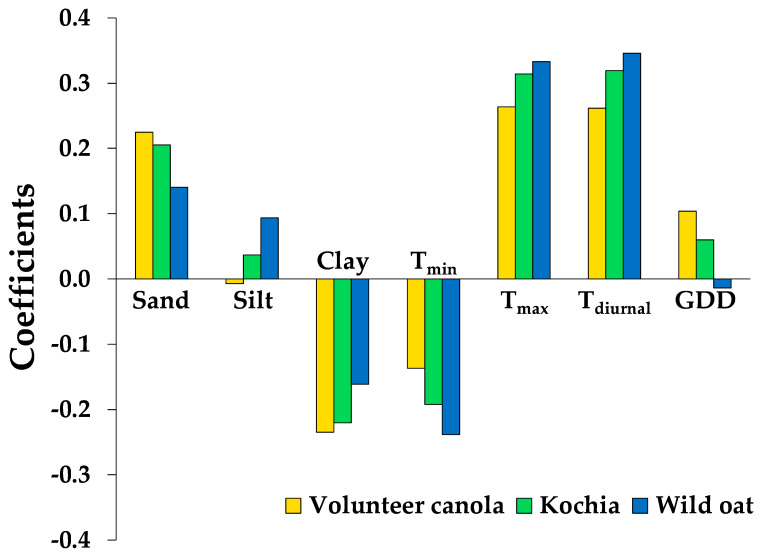
Parameter estimates from the partial least squares analysis retaining only influential independent variables (Variable Importance for the Projection statistic ≥0.8). The coefficients indicate the association of each independent variable with seed survival of the weed species. Abbreviations: T_min_, T_max_, and T_diurnal_ indicate the mean daily minimum, maximum, and diurnal range, respectively, in soil temperature throughout the 7 week bioassay; GDD indicates cumulative growing degree days at 10 cm soil depth (T_base_ = 0 °C).

Weed seed survival in the soil seedbank was associated with soil physical properties and seed microsite characteristics, but not soil chemical properties. The two factors extracted during PLS analysis explained 63% and 89% of the variability in the dependent (weed seed survival) and independent variables (soil physical properties and seed microsite characteristics), respectively (data not shown). The PLS analysis revealed that soil sand (ranging among environments from 34% to 62%) and silt (29–35%) contents, the mean daily maximum soil temperature (T_max_, 23.7–31.9 °C), diurnal temperature range (T_diurnal_, 5.8–20.4 °C), and thermal time (1053–1483 cumulative GDD) over the 7 week burial period were associated positively with weed seed survival (Table 2; Figure 3). In contrast, soil clay content (8–34%) and mean daily minimum temperature over the 7 week burial period (T_min_, 11.3–17.9 °C) were associated negatively with seed survival (Table 2; Figure 3). Crop species, soil OM, pH, soluble salts, NO_3_-N, P, K, SO_4_-S, and the mean daily average temperature over the 7 week burial period (T_avg_) were not associated with weed seed survival (VIP statistic < 0.8) (data not shown). Soil silt content and seed microsite temperature dynamics (T_max_, T_min_, and T_diurnal_) were associated to the greatest extent with seed survival of wild oat, followed by kochia, and volunteer canola (Figure 3). Conversely, soil sand and clay contents and cumulative thermal time near the seed microsite had a greater impact on volunteer canola seed survival, followed by kochia and wild oat (Figure 3). In general, the direction of association of soil physical properties and seed microsite characteristics with weed seed survival remained consistent among the weed species, with few exceptions (Figure 3). 

## 4. Discussion

Our results correspond with previous studies showing wide variability in seedbank persistence among burial environments [32,38,39]. For example, giant foxtail (*Setaria faberi* Herrm.), common lambsquarters (*Chenopodium album* L.), and velvetleaf (*Abutilon theophrasti* Medik.) seedbank persistence ranged among environments from 7–42%, 5–95%, and 5–88%, respectively, after one year of burial in a multi-site-year study across the U.S. corn belt [32]. Differences in soil type among burial environments likely contributed to the variability observed in seed survival in the current study (Figure 2). In a common garden study of nine soils in western Canada, volunteer canola seed survival after 5 months of burial over winter was greater among the clay soils compared with the sandy loam and loamy sand soils, whereas the opposite relationship was observed over summer [13]. A similar response was observed after six months of oilseed rape (*Brassic napus* L.) burial over winter, where seed survival was greater in a clay and a silty clay loam soil compared with a sandy loam soil [40]. In another soil common garden study in Australia, no sterile oat (*Avena sterilis* L.) seeds were recovered after 9 months of burial in a silty loam soil, whereas intact seeds were recovered up to 21 months after burial in a sandy loam and light clay; the full duration of their burial experiment [41]. Greater survival (lower mortality) of annual ryegrass (*Lolium rigidum* Gaud.) seed was observed up to 8 months following burial in a sandy loam compared with a clay soil [42]. The soils in the current study spanned sandy loam, loam, and clay loam textures (Table 1); however the impact of these soils on weed seed survival was represented as part of the burial environment effect. Because conditions of the current study correspond with a burial period during the summer growing season, our results correspond with previous observations [13], where clay content was associated negatively and sand content associated positively with volunteer canola seed survival over summer (Figure 3).

Although soil texture in a broad context has been shown to impact weed seed survival in the soil seedbank [13,40,41,42], less consensus has been reached regarding the impact of individual soil properties. Soil bulk density and temperature were associated strongly with volunteer canola seed survival over winter, whereas summer seedbank persistence was driven by a combination of soil texture, bulk density, OM, cation exchange capacity, and soluble salt concentration [13]. Long et al. [41] attributed differences in seed persistence of swan plant (*Gomphocarpus physocarpus* E. Mey.), sterile oat, and broadleaf privet (*Ligustrum lucidum* W.T. Aiton) among soils with different texture to soil-mediated temperature and moisture dynamics rather than texture per se. Indeed, greater soil moisture results in greater wild oat seed mortality [43], whereas dry conditions induce secondary dormancy in canola seeds and prolong their longevity in the soil seedbank [22,23,24]. Among environments, generalization of seed survival for 12 different species showed longer survival in soils with higher pH, lower moisture content, and lower C:N ratio [38]. Lambsquarters seedbank persistence was associated positively with clay and organic carbon content and associated negatively with bulk density and sand content [32]. Soil organic carbon and moisture could conceivably impact microbial communities, and their concomitant impact on weed seed decay in soil [44]. Fungal community composition in soils with contrasting management history was associated with giant foxtail and velvetleaf seed mortality, whereas the soil bacterial community was correlated with seed mortality of giant foxtail but not velvetleaf [45]. Nitrogen fertilization can also play a role in microbial seed decay [46], albeit some species are more susceptible to decay than others [44]. Weed seed survival was not associated with soil OM and NO_3_-N concentration in the current study; however these effects could have been masked by the impact of mesocosm fertilization at 2 and 4 weeks after planting. The positive association of thermal time with seed survival in the current study contrasts previous observations where hydrothermal time was associated negatively with seedbank persistence [32]. The reason for this contrast may be due to the relatively short burial duration observed in the current study.

The current study suggests a strong influence of burial environment on seed mortality of kochia, wild oat, and volunteer canola, and negligible impact of different crop species on soil seedbank persistence during early crop growth and development. Wide variability in seed mortality among burial environments and strong association of soil physical properties and seed microsite characteristics with weed seed survival indicates that management practices can be used to promote soil conditions that suppress weed seed survival. However, the limited nature of this preliminary assessment suggests that the influence of crop growth and development on soil seedbank persistence warrants further investigation under a wider range of parameters before it can be concluded that crop species does not impact weed seedbank persistence. For example, extending the burial period to the entire growing season may help provide a more complete assessment of potential crop-mediated impacts. Evaluation of multiple seed accessions would provide a more-robust estimate taking into consideration genetically controlled variability in dormancy, such as that observed for predisposition to secondary dormancy in canola seeds [23]. The scope of our limited assessment did not include evaluation of soil moisture and its potential association with weed seed mortality, which could have played an influential role in our observed results. In addition, burial depth can be a key driver of seedbank persistence and seed fate of these species [24,47,48,49,50]; albeit more-recent reports suggest that burial depth does not impact kochia seedbank persistence [19,20]. Evaluation of seedbank persistence over multiple burial depths, and further separation of seed fate into quiescence, dormancy, decay, predation, and lethal germination are critical to fully understanding the potential impacts of crop growth and development on weed seed longevity in the soil seedbank.

Weed seedbank persistence data tend to be underrepresented in models of weed population demography due to the cumbersome nature of soil seedbank studies. However, the dynamic nature of seeds in the soil seedbank warrants further consideration as simplified parameterization of seedbank persistence in demographic models may induce substantial error in model simulations. The current study suggests that weed seed mortality in the soil seedbank does not differ among common crop species grown in western Canada; however, this requires further evaluation over a wider range of parameters. Instead, these results strongly suggest consideration for demographic models to include the effects of soil and environmental variability on weed seedbank persistence. Burial environment clearly influences weed seed mortality, and further research is warranted to fully understand how environmental and edaphic factors impact seed fate of different weed species in the soil seedbank.

## Figures and Tables

**Table 1:** Description and location of soil collection and seed burial for each repetition of a 7 week bioassay assessing the impact of crop species on seed mortality of kochia, wild oat, and volunteer canola ^a^.

	Soil Collection Location				Experiment Location		
Burial Environ.	Latitude	Longitude	Soil Texture	Previous Vegetation	City, Province	Growth Environ. ^b^	Direct Sunlight
	° N	° W					h day^−1^
1	49.70	−112.69	CL	Wheat (*Triticum aestivum* L.)	Lethbridge, AB	Outdoors	7
2	49.69	−112.76	CL	Wheat (*Triticum aestivum* L.)	Lethbridge, AB	Indoors	3
3	49.69	−112.76	CL	Canola (*Brassica napus* L.)	Lethbridge, AB	Outdoors	4
4	49.33	−123.05	SL	Buttercup (*Ranunculus* spp.)	Vancouver, BC	Outdoors	8
5	50.74	−119.24	SL	Alfalfa (*Medicago sativa* L.)	Salmon Arm, BC	Outdoors	7
6	54.17	−113.00	CL	Wheat (*Triticum aestivum* L.)	Redwater, AB	Outdoors	8
7	49.70	−112.70	L	Soybean [*Glycine max* (L.) Merr.]	Lethbridge, AB	Outdoors	7

^a^ Abbreviations: CL, clay loam; Environ., environment; h, hours; L, loam; SL, sandy loam. ^b^ One bioassay experiment was conducted indoors due to outdoor space limitations, resulting in lower time in direct sunlight.

**Table 2:** Soil edaphic factors and seed microsite characteristics for each of seven burial environments included in a 7 week bioassay assessing the impact of crop species on seed mortality of kochia, wild oat, and volunteer canola ^a^.

Burial Environ.	Sand	Silt	Clay	OM	NO_3_-N	P	K	SO_4_-S	pH	Soluble Salts	T_min_	T_max_	T_avg_	T_diurnal_	GDD
	%				kg ha^−1^					dS m^−1^	°C				
1	36	34	30	3.7	7	38	1310	2	7.4	0.4	11.3	29.0	19.0	17.8	1263
2	37	29	34	3.8	16	124	1128	6	7.6	0.4	17.9	23.7	20.4	5.8	1483
3	34	35	31	3.7	20	124	1501	6	7.6	0.5	11.3	31.7	19.7	20.4	1053
4	62	30	8	10.9	7	7	166	23	5.6	0.2	17.5	25.1	20.7	7.6	1471
5	62	30	8	2.8	33	157	355	14	6.5	0.2	15.1	30.1	20.3	15.0	1476
6	41	31	28	7.6	34	160	590	54	7.3	0.6	12.5	31.9	19.9	19.4	1394
7	40	34	26	3.3	12	15	858	4	7.5	0.3	13.7	24.3	18.5	10.5	1268

^a^ Abbreviations: Environ., environment; OM, organic matter; T_min_, mean daily minimum soil temperature throughout the 7 week bioassay; T_max_, mean daily maximum soil temperature throughout the 7 week bioassay; T_avg_, mean daily average soil temperature throughout the 7 week bioassay; T_diurnal_, mean temperature variation between the daily maximum and minimum soil temperatures throughout the 7 week bioassay; GDD, cumulative growing degree days (Soil T_base_ = 0 °C) throughout the 7 week bioassay.

## Data Availability

The data presented in this study are available on request from the corresponding author.

## Supplementary Materials

The following are available online at https://www.mdpi.com/article/10.3390/plants10091961/s1, Table S1: Methods used to assess soil edaphic factors.
