# Supplementary material for: Burial Environment Drives Seed Mortality of Kochia (Bassia scoparia), Wild Oat (Avena fatua), and Volunteer Canola (Brassica napus) Irrespective of Crop Species
Source: Plants (Basel). 2021 Sep 20;10(9):1961. doi: 10.3390/plants10091961 (PMC8468733; doi:10.3390/plants10091961)
Supplement: Supplementary file 1 [file plants-10-01961-s001.zip › plants-1395650-supplementary.pdf]

plants

MDPI

**Table S1.** Methods used to assess soil edaphic factors.

| Measurement | Extraction Method | Detection Method |
| --- | --- | --- |
| Soil Texture | Sodium Hexametaphosphate | Hydrometer, 6 hrs with Gravimetric Sand Sieve |
| Organic Matter | N/A | Loss on Ignition at 375°C for 16 hrs |
| pH, EC | 1:2 Soil:Water | Probe |
| $NO_3$-N | 0.01 M $CaCl_2$ | Cd Reduction on Auto Flow Colorimetry |
| P | Modified Kelowna | Auto Flow Colorimetry |
| $SO_4$-S | 0.01 M $CaCl_2$ | ICP-OES[a] |
| K | 1 N Neutral Ammonium Acetate | Atomic Absorption |

[a] Inductively coupled plasma with optical emission spectroscopy.
